# Role of oxidative stress in impaired type II diabetic bone repair: scope for antioxidant therapy intervention?

**DOI:** 10.3389/fdmed.2024.1464009

**Published:** 2024-10-14

**Authors:** Pui Li, Kuraym Khalid Kuraym Alenazi, Jordanna Dally, Emma Louise Woods, Rachel Jane Waddington, Ryan Moseley

**Affiliations:** ^1^ Disease Mechanisms Group, School of Dentistry, College of Biomedical and Life Sciences, Cardiff University, Cardiff, United Kingdom; ^2^ Biomaterials Group, School of Dentistry, College of Biomedical and Life Sciences, Cardiff University, Cardiff, United Kingdom

**Keywords:** bone repair, type II diabetes mellitus, hyperglycaemia, mesenchymal stromal cell, osteoblast, angiogenesis, oxidative stress, antioxidant

## Abstract

Impaired bone healing is a significant complication observed in individuals with type 2 diabetes mellitus (T2DM), leading to prolonged recovery, increased risk of complications, impaired quality of life, and increased risk of patient morbidity. Oxidative stress, resulting from an imbalance between the generation of reactive oxygen species (ROS) and cellular/tissue antioxidant defence mechanisms, has been identified as a critical contributor to the pathogenesis of impaired bone healing in T2DM. Antioxidants have shown promise in mitigating oxidative stress and promoting bone repair, particularly non-enzymic antioxidant entities. This comprehensive narrative review aims to explore the underlying mechanisms and intricate relationship between oxidative stress, impaired bone healing and T2DM, with a specific focus on the current preclinical and clinical evidence advocating the potential of antioxidant therapeutic interventions in improving bone healing outcomes in individuals with T2DM. From the ever-emerging evidence available, it is apparent that exogenously supplemented antioxidants, especially non-enzymic antioxidants, can ameliorate the detrimental effects of oxidative stress, inflammation, and impaired cellular function on bone healing processes during uncontrolled hyperglycaemia; and therefore, hold considerable promise as novel efficacious therapeutic entities. However, despite such conclusions, several important gaps in our knowledge remain to be addressed, including studies involving more sophisticated enzymic antioxidant-based delivery systems, further mechanistic studies into how these antioxidants exert their desirable reparative effects; and more extensive clinical trial studies into the optimisation of antioxidant therapy dosing, frequency, duration and their subsequent biodistribution and bioavailability. By enhancing our understanding of such crucial issues, we can fully exploit the oxidative stress-neutralising properties of these antioxidants to develop effective antioxidant interventions to mitigate impaired bone healing and reduce the associated complications in such T2DM patient populations.

## 1 Introduction

Bone repair is the physiological process that occurs at sites of lost tissue, with the ultimate aim of re-establishing normal bone structure and function (1). The various stages of bone healing are clinically important in the fields of dentistry and orthopaedics, as these are responsible for achieving the successful healing of bone defects caused by trauma, bone diseases (such as osteonecrosis and tumours), or surgical procedures that involve bone manipulation, including dental extractions, implant placement or bone augmentation techniques (2–7). Therefore, it is important to create and maintain a favourable environment to allow optimal healing at the area of bone injury or defect.

However, despite being a highly organised process, it is recognised that mechanisms underlying bone repair can be significantly disrupted or impaired by the local tissue microenvironment, including via metabolic, cellular and molecular changes induced through the uncontrolled glycaemic control and hyperglycaemia associated with type 2 diabetes mellitus (T2DM). T2DM is a chronic metabolic disorder characterised by insulin resistance and elevated blood glucose levels (8). T2DM and its associated patient co-morbidities represent major medical and public health concerns, due to their ever-increasing global prevalence. Indeed, T2DM is estimated to affect approximately 451 million people worldwide, with projections expecting rises to 693 million by 2045 (9). Consequently, such clinical situations provide significant economic burdens to healthcare providers. In this regard, uncontrolled T2DM is recognised as a mediator of disordered bone metabolism and homeostasis, being associated with various complications including delayed or compromised bone formation following trauma or surgical intervention, resulting in prolonged recovery time, non-union, heightened risk of other post-injury complications and reduced functional outcomes (10–12). Thus, T2DM is now an established risk factor for the development or exacerbation of dental/orthopaedic fractures, periodontal disease and implant failure (13–15), whilst normal glycaemic control is imperative to the success of these reparative processes.

Concurent with impaired bone healing in T2DM patients are several mechanisms implicated in causing dysfunctional cellular behaviour and osteogenic activities, with one of the most well-established mediators of these disrupted processes being oxidative stress (16–21). However, despite oxidative stress being widely acknowledged as a key influential factor on the various cell types involved in normal bone repair responses, our current knowledge and understanding of the roles and therapeutic potentials of the antioxidants responsible for counteracting the elevated levels and deleterious effects of oxidative stress in biological systems, including bone, remains much less in comparison. Therefore, this review article aims to explore the existing scientific preclinical and clinical evidence to provide a much needed and detailed overview of our current understanding of the mechanistic roles which oxidative stress plays in mediating altered cell signalling pathways leading to dysfunctional cellular repair responses in T2DM bone, coupled with the evidence available to support or discount the potential use of antioxidants as novel therapeutics for the alleviation of such impaired T2DM-associated healing outcomes in future.

## 2 Cellular and molecular mechanisms of normal bone repair

**Figure 1:**
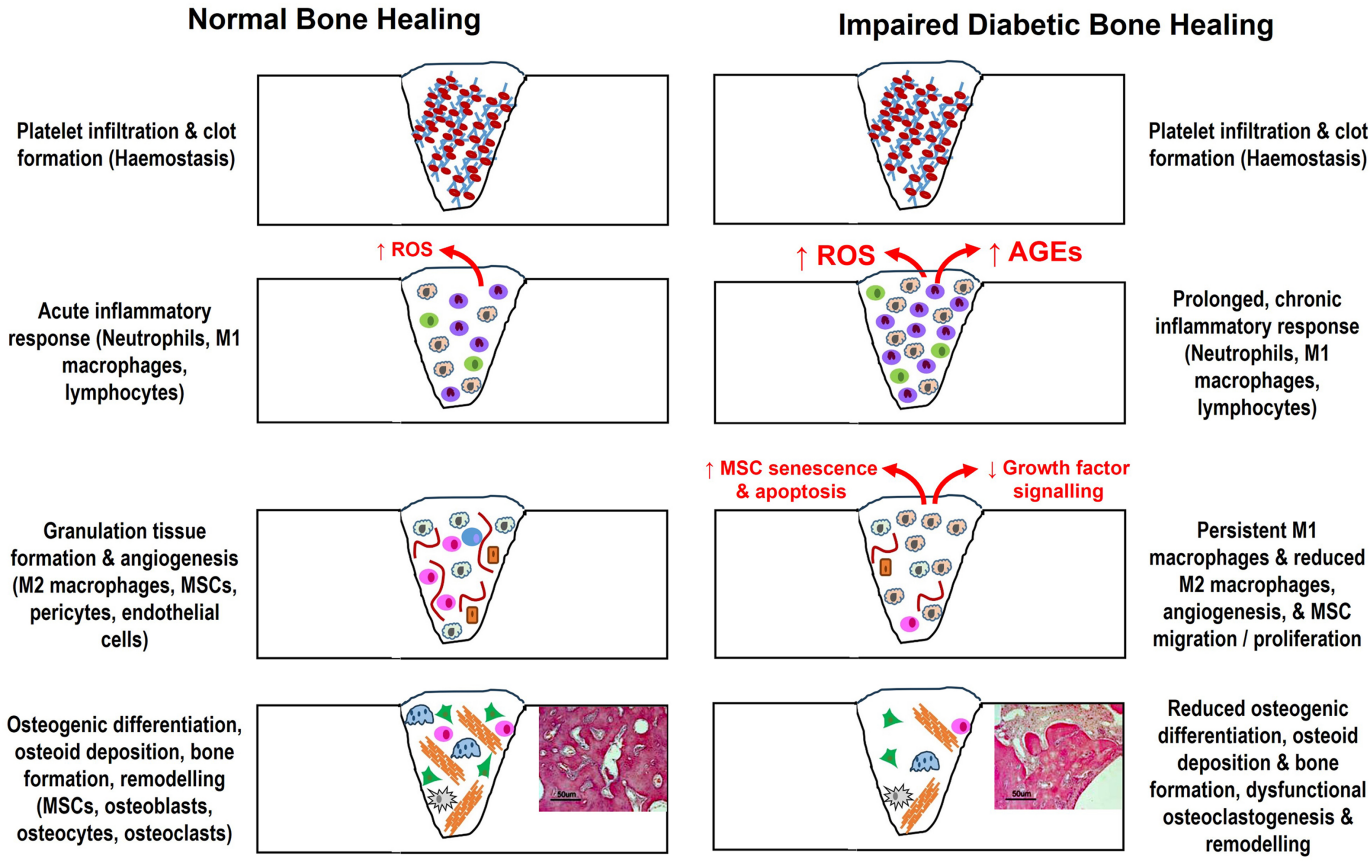
Summary of the various overlapping phases and cell types associated with bone repair to re-establish normal bone architecture and function, and how these cellular and molecular events are disrupted by the uncontrolled hyperglycaemia associated with T2DM to cause impaired healing. AGEs, advanced glycation end products; MSCs, mesenchymal stromal cells; ROS, reactive oxygen species.

Bone healing is regarded as a complex, but tightly organised process, consisting of several highly coordinated overlapping phases, involving inflammation, repair and remodelling; mediated through cooperation between various cell types and intracellular/extracellular signalling molecules to re-establish normal bone architecture and function [(1, 22–25); summarised in Figure 1]. Bone repair commences when a bone is injured, leading to vascular disruption. Platelet-mediated fibrin clot formation occurs during haemostasis and acts as a provisional matrix, releasing chemotactic growth factors and pro-inflammatory cytokines, such as interleukin-1 (IL-1), IL-6 and tumour necrosis factor-α (TNF-α) (23). Subsequently, inflammation occurs, leading to the recruitment of neutrophils, classical pro-inflammatory M1 subtype macrophages and lymphocytes to the wound site (24–27). These inflammatory cells are primarily responsible for the removal of bacteria, minimising the risk of wound site infection.

Following inflammation, the blood clot transitions into granulation tissue, where mesenchymal stromal cells (MSCs) are recruited to the wound site from the bone marrow and periosteum; along with alternative anti-inflammatory M2 subtype macrophages, responsible for removing cellular debris and the promotion of angiogenesis, facilitated via production of a wide range of anti-inflammatory mediators as healing progresses (24–26). Such important roles for MSCs in bone healing are attributed to their self-renewal properties and responses to growth factors, which initiate migratory and proliferative responses, in addition to their osteogenic lineage differentiation commitment to form mature osteoblasts via upregulation of transcription factors, such as Runx2 and Osterix. Osteoblasts are responsible for initiation of extracellular matrix (ECM) synthesis, deposition, remodelling and subsequent mineralisation (22, 23). Pericyte recruitment is also essential to stimulate endothelial cell formation; whilst neo-angiogenesis and vascular ingrowth support tissue repair via supplementation of nutrients and additional MSCs to wound sites, both of which are necessary for osteogenesis to occur (28, 29).

Central to these osteogenic and angiogenic responses at wound sites are the upstream activation of cell signalling pathways, principally orchestrated through the stimulation of endogenous connective tissue cells by a plethora of growth factors, including transforming growth factor-β_1_ (TGF-β_1_), bone morphogenetic proteins (BMPs) and vascular endothelial growth factor (VEGF) (30–32). Such growth factors possess osteoinductive, osteoconductive and osteoadaptive properties that instigate ossification. Maintenance of MSC populations is achieved through the presence of niches, located within various bone regions (33, 34).

During bone repair, MSCs are primarily sourced from two distinct niches within the bone marrow cavity (35). These include the highly vascularised perivascular/sub-endosteal niche, which contains endothelial cells, hematopoietic stem cells (HSCs) and uncommitted MSCs (36–38); whereas the endosteal niche, located at the interface between trabecular bone and bone marrow, comprises uncommitted MSCs, pre-osteoblasts and osteoblasts lining compact bone (39–41). It was originally proposed that bone marrow-derived MSCs are the main source responsible for bone repair. However, it has since been shown that MSCs within both the perivascular and endosteal niches possess important roles in facilitating bone repair processes overall (34, 35). Indeed, committed lineage restricted MSCs lining the endosteum also play key roles in mediating bone repair responses, acting as “first responders” during mineralised tissue repair (41–43).

MSC osteogenic differentiation into mature osteoblasts is followed by the synthesis and secretion of bone osteoid, an immature bone ECM consisting of type I collagen, proteoglycans, such as the small leucine-rich proteoglycans (SLRPs), decorin and biglycan; and various bone glycoproteins (such as osteocalcin, osteonectin, osteopontin and bone sialoprotein), which possess distinct roles in regulating normal matrix-mediated mineralisation (1, 37–39). Herein, some osteoblasts become embedded within the mineralised ECM to form osteocytes, with roles in regulating bone homeostasis and osteoblast/osteoclast formation via hormonal and mechanical cues; whilst others undergo apoptosis to arrest further bone synthesis (44, 45).

The latter phase is tissue remodelling involves replacement of immature woven bone with mature lamellar bone over time, driven by mechanical loading and mediated via the coordinated action of osteoblasts and bone resorbing osteoclasts (1, 37, 39, 46–50). Osteoblasts regulate osteoclastic differentiation from HSCs, via secretion of receptor activator of nuclear factor-kappa B ligand (RANKL) and osteoprotegerin (OPG), which control RANKL interaction with receptor activator of nuclear factor-kappa B (RANK) on HSC surfaces and osteoclast formation overall. Hence, the type and quality of bone formed not only relies on the tissue and anatomical location of the wound, but also the mechanical conditions in the wound site (38, 47, 50).

## 3 Type 2 diabetes mellitus and impaired bone healing

Due to the varied mineralised tissue perturbations observed in T2DM patients, extensive preclinical studies have been performed to enhance our understanding of the cellular, molecular and metabolic events that support delayed/dysfunctional bone formation and the macro/microscopic changes in bone architecture associated with T2DM, such as deteriorations in composition, volume, quality and biomechanical properties. Indeed, it is known that T2DM influences bone quantity properties, such as the relative mineral density and porosity, which significantly affect overall bone quality. Consequently, various biomechanical analyses have shown that diabetic bone derived from humans and rodent model studies are accompanied by increased fragility and fatigue, with reductions in crucial parameters, such as mechanical load, elasticity, energy absorption and stiffness (51–55).

Impaired bone healing observed in individuals with T2DM is a multifactorial phenomenon, affecting all stages of bone repair and mediated by numerous initiators, including hyperglycaemia, chronic inflammation, oxidative stress, advanced glycation end products (AGEs), polyol pathway, high protein kinase C (PKC) activity and hexosamine biosynthesis pathways; which abrogate bone healing and angiogenic processes [(8, 10–15); summarised in Figure 1]. Chronic inflammation, which is commonly observed in T2DM, further exacerbates the impairment of bone healing processes, although it has been proposed that the chemotactic ability to recruit inflammatory cells to wound healing sites declines, compared to normo-glycaemic environments. The diabetic bone environment is further associated with a delayed, but sustained increase in the secretion of pro-inflammatory cytokines, such as IL-1β and TNF-α, capable of inhibiting osteoblast differentiation and activity, but activating osteoclast formation and bone resorption (46, 49). Furthermore, dysregulated immune responses and the prolonged presence of pro-inflammatory M1 macrophages during T2DM, at the expense of reparative M2 macrophages, may lead to delayed resolution of inflammation and compromised tissue repair (56–63).

Additional studies have suggested that impaired diabetic bone healing is due to the delayed onset of osteogenic responses during T2DM, culminating in histopathological features such as osteopenia and decreased bone formation *in vivo* (56, 63–67). Hyperglycaemia is well-established to significantly impact normal bone marrow-derived MSC and osteoblast responses, which exhibit reduced proliferative capabilities due to shortened telomeres and early-onset replicative senescence, negatively influencing MSC viability and apoptosis, colony-forming efficiency, multi-potency and osteogenic differentiation capabilities overall (68–74). Impaired osteogenesis under hyperglycaemic conditions has been reported to occur due to preferential MSC adipogenic differentiation, driven by peroxisome proliferator-activated receptor-γ (PPAR-γ) (69, 71, 75).

Despite many studies supporting the detrimental effects of hyperglycaemia on MSC and osteoblast responses in bone, other studies suggest limited effects of hyperglycaemia on MSC and osteoblast functions (76–79). These inter-study variations are a proposed consequence of factors, such as the varied MSC isolation procedures implemented, the source, purity and heterogeneity of isolated MSC populations, and the contrasting glucose concentrations/exposure periods used during *in vitro* studies (41). Indeed, despite most *in vitro* hyperglycaemia studies demonstrating significant deleterious effects of hyperglycaemic conditions on the proliferation and osteogenic differentiation of human or rodent MSC responses derived from the perivascular niche (68–74), recent evidence suggests that hyperglycaemia has limited impact on the proliferative and stem cell characteristics of MSC populations derived from the endosteal niche of compact bone (39–43, 80). However, endosteal niche-derived MSCs are susceptible to reduced osteogenic and adipogenic differentiation capabilities under such hyperglycaemic conditions.

In addition to direct effects of uncontrolled glucose levels on MSC and osteoblast wound healing responses, the diabetic bone microenvironment can further disrupt cellular reparative functions via dysregulation of growth factor signalling and alterations in bone ECM composition, both of which have further repercussions for bone repair overall. Despite various growth factors exhibiting essential roles in the regulation of normal bone repair (30–32), T2DM induces an imbalance in the expression and signalling of many growth factors, including TGF-β_1_, BMPs, VEGF, fibroblast growth factor-2 (FGF-2) and insulin-like growth factor-1 (IGF-1), which disrupts their bioavailability and cell signalling mechanisms, contributing to dysregulated angiogenesis and bone healing overall.

The delayed, but sustained secretion of TGF-β_1_ have been reported, associated with high glucose-treated MSCs and other *in vivo* models of diabetic bone repair, in addition to sera derived from T2DM patients (56, 63, 73, 81–84). Such elevated TGF-β_1_ levels have been shown to be predominantly derived from MSCs following short-term exposure to hyperglycaemia, whilst prolonged high glucose exposure significantly retarded TGF-β_1_ expression and secretion by MSCs (63). Both scenarios could have severe consequences for normal bone repair, as high TGF-β_1_ levels are known to be inhibitory towards osteoblast differentiation and ECM deposition during the latter stages of osteogenesis (85–88); whilst reduced TGF-β_1_ levels with long-term glucose exposure could result in complete attenuation of osteogenic differentiation by MSCs and subsequent bone ECM deposition and mineralisation (31, 32). Similar profiles have also been shown with BMP-2, BMP-4 and BMP-6, where delayed expression, followed by subsequent elevated levels and sustained disruption to the normal secretion and cell signalling profiles have been reported (83, 89–93). Decreased VEGF, FGF-2 and IGF-1 levels have further been associated with diabetic bone healing, which impedes both osteogenic and angiogenic responses within the healing tissue (55, 92–96).

Such direct effects on growth factor signalling and MSC osteogenic differentiation further impact on the deposition and remodelling of the bone ECM, with significant perturbations in composition and subsequent mineralisation reported. A prominent feature of impaired diabetic bone healing is decreased type I collagen synthesis. In combination with the established structural changes to collagen molecules, due to AGEs forming non-enzymatic irreversible crosslinks within the collagen triple helix and between adjacent fibres, normal collagen enzymatic crosslinking processes are compromised, resulting in impaired bone stability, strength and quality (97–101). As these AGE-based crosslinks impede the proteolytic remodelling of type I collagen by osteoclasts, their reduced turnover leads to an accumulation of abnormal type 1 collagen that contribute to diminishing biomechanical properties in diabetic bone (102).

In addition to type I collagen, the expression, protein levels and structural properties of various non-collagenous ECM components are further altered during T2DM. Regarding the prominent proteoglycans within bone, decorin and biglycan, recent studies have demonstrated that MSC expression and secretion are enhanced following short-term hyperglycaemic exposure (63, 102). However, prolonged exposure to high glucose conditions significantly arrested decorin and biglycan expression and protein levels. Numerous glycoproteins commonly localised within bone are also influenced by hyperglycaemic conditions, largely exhibiting altered expression of osteocalcin, osteopontin and bone sialoprotein *in vitro*, with delayed and/or sustained levels identified *in vivo* (56, 68, 81, 85, 103–107). Such ECM component dysregulation has collectively been proposed to perturb the sequence and orchestration of events that occur during normal bone mineralisation and repair, such as collagen fibrillogenesis, mineral deposition, crystal growth, TGF-β_1_ bioavailability and osteogenic cell signalling; further contributing to delayed or impaired bone healing overall (108–110).

There is also substantial evidence confirming that the uncontrolled glycaemic control associated with T2DM disrupts normal endothelial cells functions and neo-angiogenesis, essential for oxygen and nutrient provision and successful bone repair (28, 29). Specifically, preclinical and clinical studies have demonstrated that hyperglycaemia induces alterations in hypoxia-inducible factor 1 (HIF-1) levels. Consequently, pro-angiogenic signalling pathways are abrogated, resulting in decreased VEGF expression, as well as that of other markers, such as PECAM-1; whilst promoting the activation of anti-angiogenic signals (55, 93, 97, 107, 111, 112). Additionally, endothelial progenitor cells (EPCs) within the bone marrow vascular niches are significantly reduced in number by hyperglycaemia (113, 114). Such cellular losses lead to impaired EPC and microvascular endothelial cell functions, via reduced proliferation, migration and tube formation, together with increased autophagy and apoptosis, in part mediated via transcription factor, FOXO1; and culminating in defective revascularisation (61, 112, 114–118).

Due to such hyperglycaemic and pro-inflammatory environments, osteoblast differentiation and functions are significantly disrupted during T2DM. Such events may be abrogated further due to increased RANKL and reduced OPG expression, in favour of enhanced HSC differentiation, osteoclast formation and bone resorption (104, 119, 120). That said, the role of osteoclastogenesis in T2DM is quite contentious, as there is conflicting evidence to suggest opposing low RANKL and high OPG expression profiles in osteoblasts under high glucose conditions, potentially limiting bone resorptive activities (74, 121). Similarly, despite hyperglycaemia leading to aberrant osteoclast differentiation and increased bone resorption, *in vitro* experiments suggest that changes in bone architecture typical for T2DM are not a direct consequence of excessive bone resorption (74). As it has also been proposed that osteoclasts possess decreased bone resorption activity under hyperglycaemic conditions (107, 122), it is plausible that both osteogenesis and osteoclastogenesis are impaired during T2DM.

Hence, the impairment of normal osteoblast and osteoclast reparative responses, coupled with dysfunctional inflammation and attenuated angiogenic responses that compromise blood supply to the wound site, collectively lead to delayed healing and reduced bone formation during T2DM. Consequently, understanding the intricate relationship between oxidative stress, impaired bone healing and T2DM, is crucial for developing targeted therapeutic strategies to improve bone repair outcomes in diabetic individuals. Therefore, the remainder of this narrative review focusses on providing a comprehensive analysis of the mechanisms underlying impaired bone healing associated with T2DM, with a specific emphasis on the role of oxidative stress and potential interventions involving antioxidants. By targeting these underlying oxidative mechanisms and restoring the redox balance between reactive oxygen species (ROS) production and antioxidant defences, it may be possible to develop novel therapeutic strategies that address the altered inflammatory, angiogenic and osteogenic responses, to restore bone healing and mitigate the clinical complications associated with T2DM.

## 4 Oxidative stress and antioxidants

Oxidative stress, referring to an imbalance between ROS production and cellular/tissue antioxidant defence mechanisms, plays a pivotal role in the pathogenesis of numerous diseases, including T2DM and impaired bone healing (16–21). ROS, such as superoxide radicals (O_2_^·−^), hydrogen peroxide (H_2_O_2_), and hydroxyl radicals (·OH), are generated as by-products of normal cellular metabolism and can be further produced by various enzymatic sources, including NADPH oxidases, xanthine oxidase, and the mitochondrial electron transport chain (16–18, 21). Additionally, in uncontrolled chronic diabetic environments, AGEs can be produced. AGEs are a diverse group of compounds spontaneously produced by non-enzymatic glycation or oxidation of various proteins, including type I collagen (97–102), and increased in T2DM patients due to hyperglycaemia and the accompanying elevations in oxidative stress (123). AGEs also promote inflammation and the production of pro-inflammatory cytokines, perpetuating impaired healing (124). Consequently, low ROS levels are purported to play important roles in regulating cell signalling and functions (125). However, excessive production of highly reactive ROS and AGE molecules can cause indiscriminate modifications and damage to cellular and ECM components, including DNA, proteins, lipids and carbohydrates, which negatively impacts normal biomolecular and cellular functions (16, 17). Furthermore, interactions between AGEs and their cell surface receptor, receptor for AGEs (RAGE), can enhance further pro-inflammatory cytokine and ROS production (123, 126), resulting in a continual cycle of chronic inflammation and bone resorption.

ROS levels are tightly regulated by enzymic and non-enzymic antioxidant defence mechanisms, which act by neutralising and scavenging ROS, maintaining redox homeostasis, and preventing cellular damage (16, 17, 19, 21, 127). Antioxidants are compounds that can neutralise or reduce the harmful effects of ROS, which can be divided into endogenous and exogenous antioxidants that help mitigate oxidative stress. Endogenous antioxidant mechanisms include enzymes, such as superoxide dismutases (SODs), catalase, and glutathione peroxidases (GPx), as well as non-enzymatic antioxidants such as glutathione.

Three distinct SOD isoforms have been identified in mammalian cells, with the copper-zinc containing SOD (SOD1) and manganese containing SOD (SOD2) being the most significant. SOD1 and SOD2 are both ubiquitously expressed in aerobic cells, being localised intracellularly within the cytosol and mitochondria, respectively (128, 129). In contrast, the copper-zinc containing SOD isoform (SOD3) possesses more restricted expression and is particularly localised within pericellular and ECM environments (128, 130). Nonetheless, wherever located, SOD isoforms have key roles in alleviating oxidative stress by catalysing the dismutation reaction to convert O_2_^·−^ to H_2_O_2_ (16, 17). Most aerobic cells also contain the haem containing enzyme, catalase, particularly localised in the cytosol within the peroxisomes (131), which detoxifies H_2_O_2_ into H_2_O (16, 17). In addition to catalase, H_2_O_2_ decomposition is aided by glutathione-metabolising enzymes, including glutathione peroxidases (GPXs), S-transferases (GSTs), reductases (GSRs) and synthetases (GSSs) (132, 133). GPXs catalyse the oxidation of reduced glutathione (GSH) to oxidised glutathione (GSSG) by H_2_O_2_ within the cytosol, with GSH synthesis being regulated and restored through the actions of GSRs and GSSs.

Enzymic antioxidants are themselves regulated at a gene level by nuclear factor E2-related actor 2 (Nrf2), a transcription factor normally located in the cytosol under basal oxidative conditions, being tightly regulated by Kelch-like ECH-associated protein 1 (Keap1), a redox-sensitive ubiquitin ligase which tethers Nrf2 within the cytosolic compartment (19, 134, 135). Under conditions of oxidative stress, Keap1 is oxidised at reactive cysteine residues, resulting in Keap1 inactivation, stabilisation of Nrf2 and subsequent translocation to the nucleus where antioxidant response element (ARE) binding and gene transcription is initiated. Notable gene targets of Nrf2 regulation include glutathione regulatory genes, such as GPx, glutathione disulfide reductase 1 (GSR1) and glutathione synthetase (GSS) (134, 135). In contrast, exogenous antioxidants are primarily comprised of dietary nutrients, including ascorbic acid, α-tocopherol, selenium and various phytochemicals, which provide additional protection against oxidative damage (16, 17).

### 4.1 Oxidative stress and impaired bone healing during T2DM

**Figure 2:**
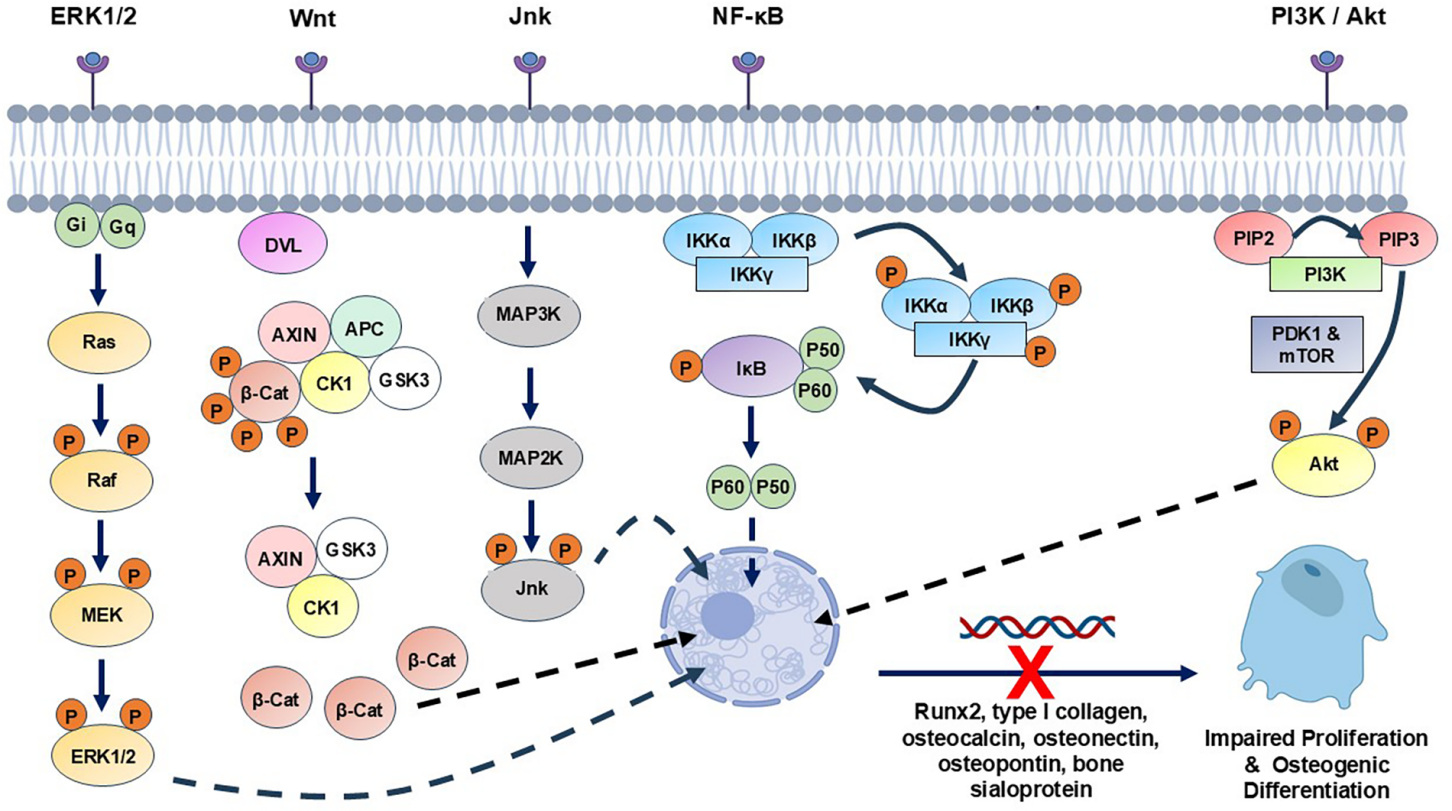
Summary of the main cell signalling pathway mechanisms reportedly disrupted by hyperglycaemia-associated oxidative stress and AGEs in MSCs, osteoblasts and osteocytes, potentially contributing to impaired bone healing in T2DM patients. ERK1/2, extracellular signal-regulated kinase; JNK, c-Jun N-terminal kinase; NF-κB, nuclear factor κB; PI3K, phosphoinositide 3-kinase.

During normal bone healing, a delicate balance exists between oxidative stress and antioxidant defence mechanisms, which permit tissue repair to proceed. Normal glucose metabolism occurs through the tricarboxylic acid cycle, involving the activation of electron pump channels and electron movement across mitochondrial membranes to generate energy. However, in individuals with T2DM, high glucose levels increase the voltage across the membranes to significantly elevate O_2_^·−^ production, disrupting the ROS/antioxidant balance (18, 21, 126, 127). This imbalance induces detrimental effects in bone cells, ECM and tissues, via disruption of cell signalling pathways involved in inflammation, angiogenesis and bone formation, exacerbating impaired healing (16–21, 123–127). In addition to elevated ROS production by neutrophils and M1 macrophages during T2DM-associated chronic inflammation (56–63), hyperglycaemia contributes to ROS overproduction through multiple pathways, including increased glucose auto-oxidation, AGE formation, PKC activation and various other signalling pathways involved in inflammation, angiogenesis and osteoblast differentiation [(16–21, 123–127); summarised in Figure 2].

Several studies have demonstrated the association between oxidative stress and impaired bone healing with T2DM. The excessive ROS generated under such diabetic states can induce direct deleterious effects in resident bone cells, including osteoblasts, osteoclasts, and osteocytes, disrupting their functions and survival. MSCs and osteoblasts are particularly susceptible to oxidative stress-induced damage and dysfunction, leading to reduced proliferation and migration, increased senescence and apoptosis, in addition to impaired MSC osteogenic differentiation and ECM synthesis (64, 136–142); thereby promoting osteopenia and compromised mineralisation overall (64, 65, 143–145).

Excessive ROS exposure is also capable of inducing comparable impairment in osteocyte activities, culminating in disrupted bone homeostasis (44, 45, 146–151). Furthermore, analogous studies involving MSC, osteoblast and osteocyte interaction with AGEs have reported similar findings (69, 73, 152–162), with responses orchestrated by altered mitochondrial function, enhanced ROS production and the induction of endoplasmic reticulum (ER) stress (69, 152, 153, 161, 162). ROS- and AGE-induced alterations in extracellular signal-regulated kinase (ERK1/2), c-Jun N-terminal kinase (JNK), p38 mitogen-activated protein kinase (MAPK), nuclear factor κB (NF-κB), Wnt and phosphoinositide 3-kinases (PI3K)/Akt signalling in MSCs, osteoblasts or osteocytes, have each been implicated in the dysregulation of normal osteogenic repair responses, bone formation and function [(136, 140, 141, 148, 154, 163, 164); Figure 2].

Although ROS and AGE effects on osteogenic differentiation would impede ECM component expression by mature osteoblasts (68, 70, 85, 97, 136, 154, 157, 158), ROS and AGEs are also capable of disrupting bone tissue architecture and mineralisation through the direct modification and degradation of ECM components commonly localised within bone. As detailed above, a large body of evidence exists to demonstrate that AGE reactions with type I collagen fibres negatively affects the biomechanical properties of bone, such as bone stability, strength and quality (97–101). However, ROS are also well-established mediators of type I collagen modification and degradation, especially the ·OH species (16). ROS exposure initially promotes a reduction in collagen gelation, increased cross-linking, aggregation, and collagen insolubility, followed by extensive degradation to low molecular weight peptides, and an increased susceptibility to proteolysis (165–168). The basis of these alterations in collagen structure is the modification and loss of functional groups of certain amino acids, such as methionine, histidine and tyrosine residues (16, 168). Considering the essential requirement to maintain the correct composition, architecture and orientation of type I collagen fibres to facilitate the initiation and progression of normal bone mineralisation within the gap zones between tropocollagen molecules (1, 37, 110), such type I collagen structural modifications would have severe implications to the propagation of bone mineralisation in T2DM patients.

Similar structural changes have also been identified in bone proteoglycans following ROS exposure. Bone proteoglycans, predominantly the SLRPs decorin and biglycan, have been shown to be susceptible to ROS-induced degradation, with degradative effects particularly manifested as amino acid modification within the core protein structure, such as leucine, proline, tyrosine and phenylalanine residues, leading to protein cleavage (16, 169). In contrast, the proteoglycan chondroitin 4-sulphate glycosaminoglycan (GAG) chains remained relatively intact, unless exposed to ·OH species (16, 169, 170). As these proteoglycans possess pivotal roles in the initiation and progression of bone mineralisation, through the regulation of collagen fibrillogenesis, mineral deposition, crystal growth, TGF-β_1_ bioavailability and osteogenic cell signalling; such manifestations could additionally impact on normal bone mineralisation events in T2DM bone (108–110, 171).

Oxidative stress has also been suggested to promote osteoclast differentiation and activity, leading to excessive bone resorption and impaired bone. Although osteoclastogenesis may be activated via the elevated levels of pro-inflammatory cytokines, such as IL-1β, IL-6 and TNF-α, associated with chronic diabetic inflammation (7, 46, 49, 56–63); most preclinical studies propose that ROS and AGEs can exert direct stimulatory effects on HSCs and osteoclasts to enhance bone resorption. Increased H_2_O_2_, ER stress, autophagy, inactivation of Nrf2 and elevated RANKL/OPG ratios, are all implicated in mediating these responses (104, 121, 151, 172–180). Such events are activated via several signalling pathways, including p38 MAPK, JNK, ERK1/2 and NF-κB, which further exacerbates bone repair (148, 177, 179). However, conflicting preclinical studies have suggested that hyperglycaemic conditions actually disrupts normal bone resorptive mechanisms in osteoclasts, via reductions in RANKL/OPG ratios, leading to dysfunctional bone turnover (74, 181, 182).

In addition to direct influences on bone cells and the ECM, there are numerous reports on the effects ROS and AGEs on immature and mature endothelial cells, and the bone vasculature, which could further induce contributory factors to delayed bone healing. Indeed, ROS and AGEs have been shown to impaired endothelial progenitor and endothelial cell responses, such as proliferation and migration, with increased apoptosis, leading to abrogated neo-vascularisation via p38 and p44/42 MAPK activation and attenuated angiopoietin-1 (Ang-1) signalling (164, 183–189).

Elevated ROS generation and oxidative stress during T2DM induce the increased detection of DNA, protein and lipid oxidative stress biomarkers associated with diabetic bone pathology and/or diminished healing (143–145, 190–192). These events are enhanced by compromised antioxidant defences, due to reduced enzymic antioxidant expression/activities and decreased levels of endogenous antioxidant capacity overall (16, 17, 19, 127–135). Indeed, the expression/activities of Nrf2, SODs, catalase and GPx have been observed to diminish in diabetic animal models and T2DM patients with impaired bone healing (143–145, 192, 193). Thus, such an antioxidant imbalance in hyperglycaemic bone would promote the uncontrolled accumulation of O_2_^·−^, H_2_O_2_ and ·OH, capable of altering normal bone healing responses by affecting cell functions and viability, the ECM and the various cell signalling pathways involved in the healing processes.

## 5 Antioxidants as potential therapies for impaired bone healing during T2DM

As it is established that antioxidant defences can be compromised in T2DM patients (143–145, 192, 193), numerous studies have investigated the potential therapeutic benefits of exogenous antioxidant supplementation, to address cellular ROS/antioxidant imbalances and improve bone healing outcomes associated with T2DM. By neutralising ROS, enhancing antioxidant defence mechanisms and/or inhibiting their damaging effects on bone and vascular cell responses, antioxidants can aid the restoration of redox balance and promote favourable conditions for bone healing overall (16, 17, 19, 127–135).

### 5.1 Enzymic antioxidants and diabetic bone repair

Despite the considerable reductions in enzymic antioxidant expression/activities accompanying impaired bone healing with T2DM (16, 17, 19, 127–135), few studies have evaluated the potential delivery of exogeneous enzymic antioxidants to hyperglycaemic bone defects, via strategies such as gene therapy, SOD/catalase and GPx mimetics (Salens and Ebselen, respectively) and nanozymes, or recombinant protein approaches, in order to alleviate oxidative stress-induced inflammation, cell/tissue damage and impaired bone healing; unlike other T2DM-related complications (194–202). Instead, as both enzymic and non-enzymic antioxidants would be expected to play essential roles in mitigating oxidative damage and promoting bone healing in individuals with T2DM, most reported antioxidant interventional studies have relied upon the application of non-enzymic antioxidants to achieve oxidative stress-counteracting outcomes.

### 5.2 Non-enzymic antioxidants and diabetic bone repair

In contrast to the status with exogenous enzymic antioxidants, considerable evidence exists supporting the promise of numerous non-enzymic antioxidant entities in improving bone healing outcomes associated with T2DM, in line with the findings of the considerable randomised controlled trials (RCTs) performed to establish the therapeutic effects of systemic antioxidant supplementation in improving insulin sensitivity, promoting glycaemic control and alleviating complications in T2DM patients, through improvements in oxidant/antioxidant status (203–205). Although not universally validated in all RCTs performed, largely due to factors associated with trial design, such as experimental group sample sizes, mono-antioxidant entity assessments, and a limited understanding of the optimal antioxidant dosing regimen (206–208), these findings still provide some support to the concept that non-enzymic antioxidant supplementation could possess therapeutic potential in diabetes management and treatment overall. Thus, it is reasonable to speculate that such systemic protective benefits would be further evident with more localised diabetic complications, including impaired diabetic bone healing. However, in contrast to the extensive number of RCTs that have previously been performed, which have shown beneficial effects of exogenous non-enzymic antioxidant supplementation on systemic complications associated with T2DM (203–205), the number of RCTs which have specifically examined the efficacies of supplementation with various non-enzymic antioxidants on bone health and healing outcomes in T2DM patients, are far fewer in comparison. Nonetheless, the findings and conclusions of these limited number of reported RCTs to date (4 in total), are summarised in Table 1.

**Table 1:** Summary of the findings of reported randomized controlled trial (RCT) studies, evaluating the efficacies of various non-enzymic antioxidants as potential therapeutics for the maintenance of bone health and healing in T2DM patients.

Antioxidant	T2DM patient sample size	Intervention	Duration	Outcomes	References
*Quercetin (Coenzyme Q)*	Total: *n = 18*. Group 1: *n = 6*; Group 2: *n = 6*; Group 3: *n = 6*	Group 1: Control (no intervention post-tooth extraction); Group 2: Application of collagen hydrogel only to extracted tooth sockets; Group 3: Application of quercetin (150 mg/mL)/collagen hydrogel to extracted tooth sockets	3 months	Supplementation with quercetin (150 mg/mL)/collagen hydrogel associated with significantly higher bone formation and density, in addition to significantly increased osteogenic marker (Runx2, osteopontin) expression in newly formed tissues.	(209)
*Resveratrol*	Total: *n = 50*. Group 1: *n = 25*; Group 2: *n = 25*	Group 1: 480 mg/day; Group 2: Placebo.	1 month	Supplementation with 480 mg/day resveratrol associated with significantly lower fasting insulin levels and insulin resistance, in addition to significantly decreased pocket depths.	(210)
*Resveratrol*	Total: *n = 192*. Group 1: *n = 65*; Group 2: *n = 65*; Group 3: *n = 62*	Group 1: 500 mg/day; Group 2: 40 mg/day; Group 3: Placebo.	6 months	Supplementation with 500 mg/day resveratrol associated with positive effects on bone density, especially in specific high-risk subgroups of patients	(211)

As the non-enzymic antioxidants suggested to exhibit bone healing efficacies under hyperglycaemic conditions are commonly derived from dietary sources, it would be rational to assume that the healthy diet and lifestyle changes advocated by clinicians for diagnosed T2DM patients would begin to address any pre-existing deficiencies in a patient's non-enzymic antioxidant profiles and total antioxidant capabilities (204, 205, 207). The principal non-enzymic antioxidants evaluated as therapeutics against impaired bone healing associated with uncontrolled hyperglycaemia and T2DM, are summarised below. Table 2 also highlights these various therapeutic interventions and key mechanistic findings from *in vitro* and *in vivo* animal model and human clinical trials.

**Table 2:** Non-enzymic antioxidants evaluated as potential therapeutics against impaired bone healing associated with uncontrolled hyperglycaemia and T2DM, and their proposed mechanisms of action based on preclinical and clinical findings.

Antioxidant & chemical structure	Effects on bone repair under hyperglycaemic conditions	Mechanisms of action/cell signalling pathways influenced	References
*Ascorbic Acid (Vitamin C)* 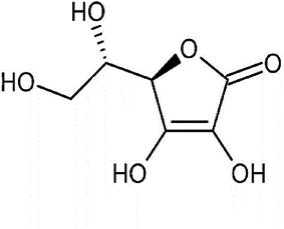	Anti-inflammatory. Enhanced MSC proliferation and osteogenic differentiation capabilities. Enhanced endothelial cell function and vascular responses. Increases bone mineral density.	Not reported.	(212–218)
*α-Tocopherol (Vitamin E)* 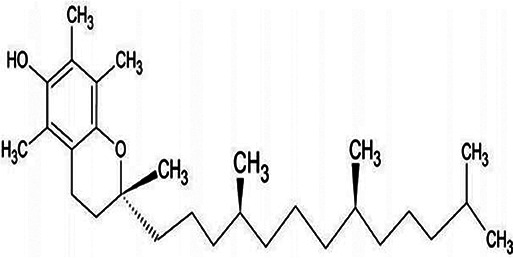	Anti-inflammatory. Reduces MSC apoptosis, whilst restoring autophagic responses. Reduces EPC apoptosis, leading to enhanced migration and angiogenic responses. Prevents alveolar bone loss in rodent models of diabetic periodontitis.	Suppresses NF-κB signalling. Activates PI3K/Akt signalling. Suppresses JNK, Notch-1, and p38 MAPK signalling.	(219–223)
*Quercetin (Coenzyme Q)* 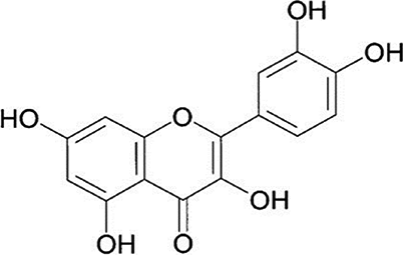	Anti-inflammatory. Reduces MSC senescence and SIRT1-induced autophagy responses. Stimulated MSC proliferation and osteogenic differentiation. Restoring EPC viability, migration, and NO^.^ and cGMP production. Improving bone mineral metabolism. Reduces bone loss, and enhances bone architecture, density and biomechanical properties.	Activates Wnt/β-catenin signalling via H19/miR-625-5p axis.	(209, 224–229)
*Resveratrol* 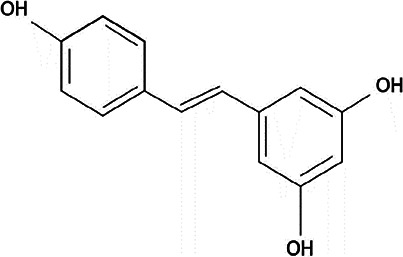	Anti-inflammatory. Reduces MSC apoptosis. Stimulates MSC osteogenic differentiation. Suppresses NOX isoform expression. Protects EPCs and endothelial cells, promoting neovascularisation responses. Reduces bone loss, and enhances bone architecture, density and biomechanical properties.	Suppresses TLR4, NF-κB, and JAK/STAT signalling. Activates Akt/GSK3β/FYN axis and Akt and E2F3 signalling. Restores SIRT1 expression. Activates Keap1/Nrf2/ARE signalling.	(210, 211, 230–243)
*Curcumin* 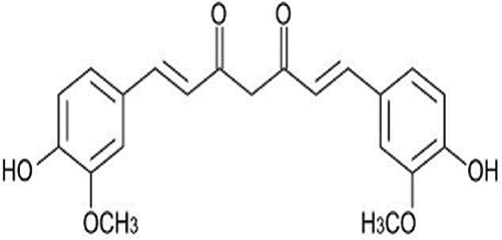	Anti-inflammatory. Stimulated MSC migration, proliferation and osteogenic differentiation. Protects EPCs and endothelial cells, promoting neovascularisation responses.	Activates TGFβ_1_/Smad2/3 signalling. Activates PI3K/Akt/NF-κB signalling.	(244–251)
*Silibinin (Silybin)* 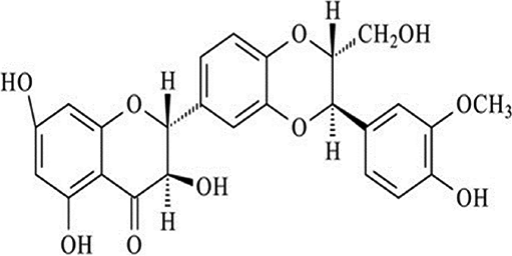	Anti-inflammatory. Protects MSCs and osteoblasts, restoring osteogenic responses. Promotes endothelial cell viability and functionality, enhancing angiogenic and autophagic responses.	Activates PI3K/Akt signalling.	(252–256)
*Coumarin* 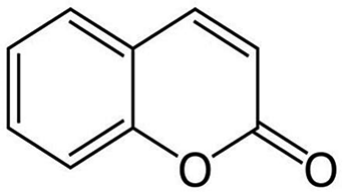	Anti-inflammatory. Promotes normal regulation of osteoclastogenesis and bone resorption by osteoblasts. Protects EPCs and endothelial cells, promoting neovascularisation responses.	Activates RANKL/OPG and RANK signalling. Suppresses AGE/RAGE signalling.	(257–260)

#### 5.2.1 Ascorbic acid (Vitamin C)

Ascorbic acid is an essential micronutrient and potent hydrophilic antioxidant, which plays a crucial role in collagen synthesis and bone formation (207, 208). Ascorbate directly scavenges ROS and subsequently protects against lipid peroxidation and protein glycation. Studies have also revealed that diabetics have lowered ascorbic acid levels, compared to their non-diabetic counterparts (207). To address such inadequacies, numerous studies have shown that ascorbate supplementation can counteract the cytotoxic effects of oxidative stress and AGEs on MSC proliferation, osteogenic differentiation, and the restoration of their paracrine signalling mechanism capabilities during hyperglycaemic conditions (212, 213); in addition to aiding the resolution of immuno-inflammatory responses in diabetic bone (214–216) and restoring endothelial cell function and other vascular responses (217). Such beneficial effects on bone reparative mechanisms are reflected in T2DM patients by the positive associations evident between high levels of circulating ascorbate and the possession of significantly greater bone mineral densities (218).

#### 5.2.2 α-Tocopherol (Vitamin E)

α-Tocopherol is another powerful lipid-soluble antioxidant, highly effective in protecting lipids from peroxidation (261, 262). α-Tocopherol has also been shown to reduce oxidative stress and inflammation via scavenging ROS and suppressing NF-κB signalling pathways (207). In contrast, reduced α-tocopherol levels are associated with the onset and development of T2DM. α-Tocopherol is well-known for influencing bone metabolism (219), demonstrating positive antioxidant and anti-inflammatory effects on MSCs derived from rodent T2DM models, especially at lower concentrations (220). Furthermore, α-tocopherol treatment reduces MSC apoptosis, whilst repairing autophagy and restoring PI3K/Akt (protein kinase B) signalling (221). Similarly, α-tocopherol reduces high glucose/hypoxia-induced cell apoptosis in EPCs, by promoting B-cell lymphoma 2 (Bcl-2) and Akt expression, and by inhibiting NF-κB p65, JNK, neurogenic locus notch homolog protein 1 (Notch-1), and p38 MAPK expression, subsequently resulting in enhanced EPC migration and increased capillary density *in vivo* (222). The elevated presence of serum α-tocopherol levels has further been shown to correlate with reductions in alveolar bone loss in rodent models of diabetic periodontitis (223).

#### 5.2.3 Quercetin (Coenzyme Q)

Quercetin is a flavonoid compound abundantly found in various fruit and vegetables, and particularly shown to be beneficial for T2DM due to its anti-hyperglycaemic and antioxidant properties (263–265). The direct ROS scavenging activity of quercetin is due to its β-ring catechol arrangement, in addition to the -OH group at position 3 of the adjoining AC rings. It is also capable of attenuating oxidative stress via the Nrf2/ARE pathway (266). Recent *in-silico* studies have further suggested that quercetin acts via the PI3K/Akt, MAPK, PKC, and JNK signalling pathways to elicit ROS-protective effects (267, 268).

Consistent with reports that quercetin possesses both anti-hyperglycaemic and antioxidant capabilities (263–265), several studies have proposed that quercetin decreases blood glucose levels and oxidative stress in rodent animal models of experimental periodontitis diabetic bone healing; improving bone mineral metabolism, serum antioxidant levels and diminishing bone loss, and thereby preventing disease progression and leading to enhanced bone architecture and biomechanical properties (224–228). At a cellular level, quercetin may facilitate bone haemostasis by limiting inflammation and by reducing senescent cells and sirtuin 1 (SIRT1)-induced autophagy. Quercetin has been shown to stimulate MSC proliferation and osteogenic differentiation, partly regulated through the H19/miR-625-5p axis to activate the Wnt/β-catenin signalling pathway (229). Furthermore, quercetin can protect EPCs from hyperglycaemia and ROS, lowering oxidative stress biomarker levels and restoring cell viability, migration responsiveness, nitric oxide (NO^.^) production, and cyclic guanosine 3′, 5′-cyclic monophosphate (cGMP) levels (269).

Due to these properties identified during preclinical studies, a RCT was performed involving quercetin delivery into tooth extraction sockets via a collagen hydrogel to promote mandibular alveolar socket augmentation post-tooth extraction, particularly prevalent with T2DM patients (209). Quercetin was shown to significantly increase bone density vs. controls, due to enhanced MSC osteogenic differentiation.

#### 5.2.4 Resveratrol

Resveratrol is a natural polyphenolic compound found in red grapes and other plant sources, which possesses both antioxidant and anti-inflammatory properties (270, 271). Resveratrol exhibits antioxidant effects by directly scavenging ROS, inhibition of NF-κB, and enhancing the antioxidant enzymes, such as SODs. These responses are largely mediated through the presence of 3′, 4′, and 5′-hydroxyl groups in its phenolic rings (272, 273). Furthermore, studies have identified that sequential proton loss electron transfer (SPLET), and hydrogen atom transfer (HAT) are the two major mechanisms underlying the direct ROS scavenging activity of resveratrol (274). Intriguingly, resveratrol has also been shown to exhibit pro-oxidant effects by activating various redox-associated signalling pathways. For instance, resveratrol promotes the production of antioxidant enzymes by activating the AMPK-FOXO1 pathway, which promotes the expression of catalase and SOD2 (275). Both Nrf2 and SIRT1 plays important roles in the protective effects of resveratrol, modulating the expression of antioxidant enzymes as well as promotes mitochondrial biogenesis, through the targeting of downstream transcription factors, including the FOXO family.

Based on these properties, there has been much interest in resveratrol from a therapeutic viewpoint in relation to the pathologies associated with T2DM, including impaired bone healing. Resveratrol has been shown to possess protective roles in ameliorating bone loss during various stages of the repair process under hyperglycaemic conditions, exhibiting anti-inflammatory effects through the suppression of pro-inflammatory cytokine levels and Toll-like receptor 4 (TLR4) and NF-κB signalling (230–232). Furthermore, resveratrol reduces MSC apoptosis and promotes osteogenic differentiation, resulting in accelerated bone healing with improved glycaemic control, bone density and trabecular architecture, and enhanced biomechanical properties (210, 211, 232–239). Amelioration of the detrimental impact of hyperglycaemia on osteogenic dysfunction has been reported to be mediated through Nrf2 activation via the Akt/glycogen synthase kinase 3β (GSK3β)/FYN axis and the restoration of SIRT1 expression in MSCs and osteoblasts (236, 237).

In addition to direct effects on osteogenesis, studies have confirmed similar protective resveratrol responses in vascular cells, with the suppression of NADPH oxidase (NOX) isoform expression coupled with the up-regulation of Keap1/Nrf2/ARE signalling and the antioxidant cascade, leading to reduced oxidative stress (240). Such antioxidant capabilities induce anti-inflammatory marker expression in endothelial cells via the down-regulation of NF-κB and JAK/STAT signalling (241), whilst further protecting and promoting neovascularisation by EPCs and endothelial cells via regulation of Akt and E2F3 signalling (242, 243).

#### 5.2.5 Other non-enzymic antioxidants with proposed diabetic bone repair capabilities

Curcumin, derived from the spice turmeric, possesses potent antioxidant, anti-inflammatory, and anti-diabetic properties (244, 245). Subsequently, studies have highlighted its beneficial effects of exogenously administered curcumin on bone healing under diabetic conditions by reducing oxidative stress, inflammation, and stimulating high quality bone formation via enhancement of MSC proliferation, migration and osteogenic differentiation, orchestrated via TGFβ_1_/Smad2/3 pathway activation (246, 247). Similarly, curcumin has been demonstrated to further bestow protective and pro-angiogenic properties to EPCs and endothelial cells, mediated through PI3K/Akt/NF-κB signalling (248–251).

Silibinin (also referred to as silybin) is a polyphenolic flavonoid present in foods, such as artichokes, which has been shown to possess potent antioxidant capabilities in various clinical situations, including those associated with T2DM (252, 253). Indeed, silibinin alleviates MSC and osteoblast dysfunction associated with oxidative stress and hyperglycaemia, by modulating PI3K/Akt signalling (254, 255). Moreover, it promotes endothelial cell viability and functionality under hyperglycaemic conditions, through its antioxidant properties enhancing angiogenic and autophagic responses (256, 276).

Coumarin is a heterocyclic compound belonging to the class of benzopyrone enriched in numerous edible plants, which possess a wide array of bioactive properties, including anti-inflammatory and antioxidant capabilities (257, 258). Hence, coumarin have been shown to ameliorate impaired bone turnover and remodelling under diabetic conditions, by promoting the expression of OPG and RANK in osteoblasts and osteoclasts, respectively, thereby restoring the normal balance between RANKL/OPG and RANK regulating osteoblast function, osteoclastogenesis and osteoclast resorptive activities (259). Coumarin has been reported to induce such effects by suppressing the interaction between AGEs and its receptor, RAGE. Such anti-AGE effects have also been identified in endothelial cells, reducing oxidative stress and inflammation (260).

Therefore, from the comprehensive range of studies performed, it is evident that such preclinical studies have largely confirmed the beneficial effects of various non-enzymic antioxidants, such as quercetin, resveratrol and curcumin, in promoting bone healing outcomes under hyperglycaemic conditions. Such studies complement recent systematic review findings of their efficacies in promoting repair during various other non-diabetic and diabetic situations associated with impaired osteogenesis (18, 228, 277). These antioxidants influence multiple signalling pathways at a cellular level to exert such diverse effects, ultimately modulating oxidative stress, inflammation, and the promotion of osteoblastic activities, leading to improved bone repair overall.

## 6 Future perspectives and implications for clinical practice

From the information above, it is irrefutable that impaired bone healing is a significant complication associated with T2DM, posing challenges for patients and healthcare providers worldwide. Recognition of the significant roles that oxidative stress and antioxidants play in impaired bone healing associated with T2DM has important clinical implications. The accumulating preclinical evidence suggesting that antioxidants, especially non-enzymic antioxidant entities, can mitigate the detrimental effects of oxidative stress, inflammation, and impaired cellular function on bone healing processes has received much attention; and holds much promise.

Despite such advances, several important considerations and challenges remain to be addressed. For instance, further studies are needed to unravel the precise cellular and molecular mechanisms underlying the effects of oxidative stress on bone cells and the specific mechanisms through which antioxidants exert their protective effects. Additionally, as inflammation, angiogenesis and new bone formation are inextricably linked during bone healing, investigating the crosstalk between oxidative stress and the other cell signalling pathways involved in the various stages of bone repair would provide a comprehensive understanding of these complex interactions. The performance of additional mechanistic studies would further help elucidate how antioxidants exert their desirable reparative effects, which would provide valuable insights into the identification of specific cellular and molecular targets for more effective therapeutic intervention development; and justification for the development of antioxidant-based therapies to improve bone healing outcomes in T2DM patients.

Although much of the preclinical evidence supporting antioxidant supplementation as a therapeutic strategy to enhance bone repair in T2DM patients has focussed upon non-enzymic antioxidants, including quercetin, resveratrol and curcumin, further research into the potential benefits of more sophisticated enzymic antioxidant-based delivery strategies should be explored. Gene therapy, SOD/catalase and GPx mimetics, nanozymes, and recombinant protein approaches show therapeutic potential in their abilities to counteract excessive oxidative stress (188–196), although the evidence supporting their effectiveness in alleviating inflammation and impaired bone healing responses associated with uncontrolled hyperglycaemia is much less in comparison. Thus, such novel interventional approaches to enhance depleted endogenous antioxidant defences accompanying impaired bone healing with T2DM (16, 17, 19, 127–135), could hold promise as future therapeutic strategies.

One significant challenge to this concept that requires major consideration for both enzymic and non-enzymic antioxidants are their pharmacological properties, including their respective mechanisms of delivery, half-life, biodistribution and bioavailability of individual antioxidant entities (199, 278, 279). Indeed, their poor bioavailability remains a significant constraint to antioxidant therapy development and efficacy, be they directly delivered locally to bone defect sites within the oral cavity, or following oral intake, gastrointestinal absorption and systemic circulation to the bone tissues, as conventionally achieved with dietary antioxidants. Thus, various antioxidant administration approaches, including both conventional and novel drug delivery systems, have been explored in attempts to enhance their pharmacological action via drug targeting and increased bioavailability. These have included numerous biomaterial- and nanomedicine-based approaches, such as 3D scaffolds, hydrogels, nanoparticles, micelles and liposomes (199, 278, 280).

From a clinical perspective, RCTs evaluating the use of non-enzymic antioxidants in impaired bone healing associated with T2DM are severely limited [(209–211), Table 1]; but show some promising results. However, in comparison, no RCTs have, to date, been performed to assess the efficacies of enzymic antioxidant-based delivery systems as novel therapeutics to address impaired healing in T2DM patients. Consequently, due to the overall lack of RCTs evaluating the potential beneficial effects of the exogenously supplemented enzymic and non-enzymic antioxidants on bone health and repair overall, there is a considerable need to address this situation in future. If successful, these studies should be subsequently progressed towards evaluations of antioxidant dosing, frequency and treatment duration, and subsequent biodistribution and bioavailability. Further comparisons of monotherapy vs. combinational antioxidant therapies, and their respective efficacies and potential synergistic effects in improving bone healing outcomes, whilst reducing the risk of complications in T2DM patients should also be prioritised. Healthcare providers involved in the care of individuals with T2DM, and impaired bone healing should consider the assessment of oxidative stress markers as part of the diagnostic and treatment process. Monitoring markers, such as ROS, oxidative damage biomarker and antioxidant enzyme levels, may help identify patients at higher risk for complications and guide the selection of appropriate interventions.

Lifestyle modifications aimed at reducing oxidative stress, such as maintaining tight glycaemic control, adopting a healthy diet rich in antioxidants, regular physical activity, and smoking cessation, could also be recommended to support bone healing processes. Furthermore, patient supplementation with exogenous antioxidants, such as ascorbic acid, tocopherol, resveratrol or quercetin, could be considered as adjunctive therapies to enhance bone healing outcomes in individuals with T2DM. However, it is important to note that the optimal dosage, treatment duration, and specific patient populations that would benefit the most from antioxidant supplementation require further investigation. Personalised treatment plans should further be developed based on individual patient characteristics, genetic factors, oxidative stress biomarker levels, response to antioxidants and T2DM severity; thereby tailoring antioxidant therapies based on patient characteristics and disease profiles that may lead to improved treatment outcomes.

While significant progress has been made in understanding the role of oxidative stress and antioxidants in impaired bone healing associated with T2DM, further studies are warranted to optimise antioxidant dosages, treatment durations, formulations and delivery methods to promote maximum efficacy and to better understand the specific mechanisms through which antioxidants modulate bone healing in the context of T2DM. Furthermore, the undertaking of comparative studies into the use of antioxidant monotherapies vs. the potential synergistic effects of combining different antioxidants as therapies or with other interventions, such as pharmacological agents or regenerative therapies should be explored, to potentially enhance bioavailability, efficacy and/or reduce side-effects. Addressing these future perspectives and challenges will enhance our understanding of the therapeutic potential of antioxidants in treating impaired bone healing associated with T2DM; and facilitate the development of safe, efficacious and accessible, antioxidant-based therapeutic interventions to improve bone healing outcomes in individuals with T2DM.

## 7 Conclusions

In summary, it is acknowledged that oxidative stress and antioxidants play critical roles in impaired bone healing associated with T2DM. Although mainly ascertained through the various preclinical studies performed, these have provided valuable insights into the potential mechanisms and therapeutic applications of antioxidants in counteracting the deleterious effects of T2DM on bone repair processes, particularly where non-enzymic antioxidants are concerned. Through a greater understanding of these mechanisms and harnessing of the potential that antioxidants can offer, we can undoubtedly aid the development of novel treatment strategies to attenuate oxidative stress and enhance bone healing outcomes in individuals with T2DM.

Exogenous antioxidant supplementation already shows promise as a therapeutic approach to mitigate oxidative stress and improve bone healing outcomes in experimental studies. However, although their ability to counteract oxidative stress, inflammation and cellular dysfunction provides a rationale for further exploration in clinical settings, additional translational studies and more comprehensive, well-designed RCTs are needed, to confirm the potential of targeted exogenous antioxidant interventions, whilst optimising dosages, treatment durations, biodistribution, bioavailability, and delivery methods to maximise their pharmacokinetic, pharmacological and therapeutic benefits; ultimately alleviating the burden of impaired bone healing and improving the quality of life in T2DM patient cohorts.
